# Ocrelizumab after natalizumab in JC-virus positive relapsing remitting multiple sclerosis patients

**DOI:** 10.1177/20552173211013831

**Published:** 2021-06-01

**Authors:** ZYGJ van Lierop, AA Toorop, EME Coerver, EAJ Willemse, EMM Strijbis, NF Kalkers, B Moraal, F Barkhof, CE Teunissen, J Killestein, ZLE van Kempen

**Affiliations:** Department of Neurology, Amsterdam Neuroscience, Amsterdam UMC, location VUmc, Amsterdam, The Netherlands; Neurochemistry Laboratory, Department of Clinical Chemistry, Amsterdam Neuroscience, Amsterdam UMC, location VUmc, Amsterdam, the Netherlands; Department of Neurology, Amsterdam Neuroscience, Amsterdam UMC, location VUmc, Amsterdam, The Netherlands; Department of Neurology, Amsterdam Neuroscience, Amsterdam UMC, location VUmc, Amsterdam, The Netherlands; Department of Neurology, OLVG, Amsterdam, the Netherlands; Department of Radiology & Nuclear Medicine, Amsterdam Neuroscience, Amsterdam UMC, location VUmc, Amsterdam, the Netherlands; epartment of Radiology & Nuclear Medicine, Amsterdam Neuroscience, Amsterdam UMC, location VUmc, Amsterdam, the Netherlands; Institutes of Neurology and Healthcare Engineering, UCL, London, United Kingdom; Neurochemistry Laboratory, Department of Clinical Chemistry, Amsterdam Neuroscience, Amsterdam UMC, location VUmc, Amsterdam, the Netherlands; Department of Neurology, Amsterdam Neuroscience, Amsterdam UMC, location VUmc, Amsterdam, The Netherlands

**Keywords:** Multiple sclerosis, natalizumab, ocrelizumab, JC virus, disease activity, progressive multifocal leukoencephalopathy

## Abstract

Ocrelizumab is often used as an alternative therapy in natalizumab-treated MS patients at risk for progressive multifocal leukoencephalopathy (PML). Our objective was to assess efficacy and safety of JC-virus positive patients switching (either directly or indirectly) from natalizumab to ocrelizumab. Forty-two patients were included from an observational cohort (median follow-up 21 months). No evidence of disease activity was found in 83% of direct switchers and 50% of indirect switchers. Two direct switchers were diagnosed with carry-over PML. Our data support a direct switch for adequate disease suppression, although carry-over PML illustrates the dilemma when choosing between a direct or indirect switch.

## Introduction

Ocrelizumab is considered an appropriate alternative therapy for relapsing remitting MS (RRMS) patients with an increased risk of natalizumab-associated progressive multifocal leukoencephalopathy (PML). Challenging aspects of switching patients from natalizumab to ocrelizumab include the choice between a direct switch or bridging with other disease-modifying therapies (DMTs) without a long-lasting pharmacodynamic effect, which can be halted more easily in case of carry-over PML.^1^ The objectives of this observational cohort study are to report on the efficacy and safety of switching JC-virus (JCV) positive MS patients from natalizumab to ocrelizumab, and to share the switch protocol of the MS Center Amsterdam.

## Methods

**Figure 1.**
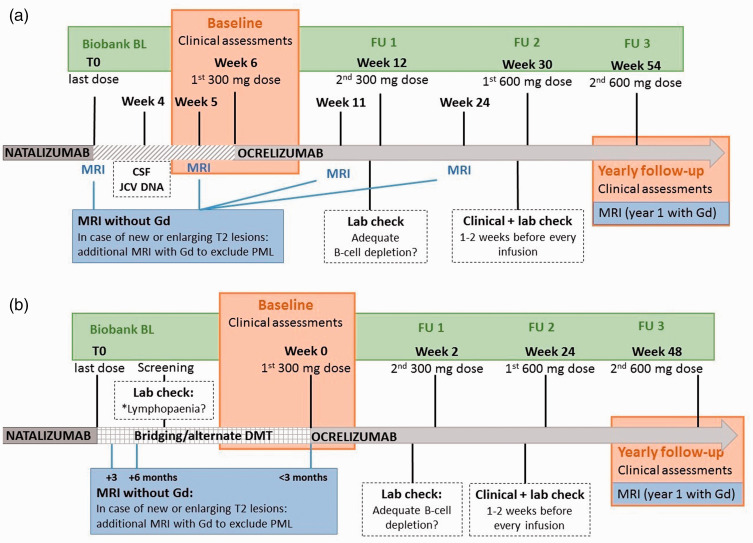
Timelines illustrating the switch protocol for JCV positive direct switchers from natalizumab to ocrelizumab (a); the switch protocol for indirect switchers who used other disease modifying therapy (DMT) in the period between natalizumab and ocrelizumab treatment (b). **A:** the first dosage of 300 mg ocrelizumab is scheduled 6 weeks after the last natalizumab dose to avoid rebound disease activity. Progressive multifocal leukoencephalopathy (PML) surveillance consists of a lumbar puncture for JCV analysis in CSF, performed 2 weeks before the first ocrelizumab and brain MRI without gadolinium (Gd), performed around the last natalizumab dose (baseline, BL) and 1 week before the first and second ocrelizumab dose. Blood samples were collected and stored at last natalizumab dose (BL), ocrelizumab screening, second 300 mg ocrelizumab dose (OCR FU1), first 600 mg dose (FU2) and second 600 mg dose (FU3). Brain MRI is repeated 3 and 6 months after the last natalizumab dose to monitor possible breakthrough of disease activity and carry-over PML. One year after BL, MRI with gadolinium is performed to evaluate efficacy of ocrelizumab. **B:** patients who use fingolimod, daclizumab or dimethyl fumaric acid switch directly to ocrelizumab, unless in the case of severe lymphopaenia the switch is extended to a maximum of 6 weeks. Brain MRI is performed 3 and 6 months after the last natalizumab dose, within 3 months prior to first ocrelizumab dose (BL) and consequently on a yearly basis. One year after BL scan, MRI with gadolinium (Gd) is performed to evaluate efficacy of ocrelizumab.

We selected all patients who switched to ocrelizumab (either directly or indirectly) because of PML risk from our prospective natalizumab cohort.^2^ The local switch protocol as implemented in the MS Center Amsterdam is presented in Figure 1(a) and (b). The decision for either a direct switch or alternative DMT was made by the treating neurologist and patient, which in some cases was driven by the availability of ocrelizumab (which came to market March 2018 in the Netherlands). Follow-up was performed from last natalizumab dose until data extraction on January 15th 2021.

Clinical evaluation during the switch was performed directly by or under supervision of the treating neurologist and included registration of relapses (defined as new neurological symptoms observed by a neurologist, lasting more than 24 hours and not attributable to other causes than MS), steroid treatment, adverse events and expanded disability status scale (EDSS) assessed by certified personnel.^3^ No evidence of disease activity (NEDA) status, defined as no relapses, no evidence of radiological disease activity and no EDSS progression (increase of 1.5, 1 or 0.5 point in case of a baseline EDSS of 0, 1-5 or ≥5.5 respectively) was assessed 6-monthly during ocrelizumab treatment. We corrected for spill-over disease activity by excluding events that occurred during or within 3 months after the switch. Our brain MRI protocol followed international guidelines.^4^

JCV indices were determined 6-monthly (STRATIFY, Unilabs, Denmark) during natalizumab and ocrelizumab treatment. These were compared to IgG levels in light of PML risk, measured at ocrelizumab screening and before every infusion. We analyzed serum neurofilament-light (sNfL) in concurrently biobanked samples using the Simoa NF-light® Advantage Kit. In SPSS for Windows version 26, we used Mann-Whitney U tests to compare between groups and Friedman’s test to compare JCV indices, IgG and sNfL levels over time. Risk factors for not achieving NEDA status after 1 year of OCR treatment (age, disease duration, duration of the switch interval between natalizumab or other DMT and ocrelizumab, relapses 1 year prior to OCR initiation, radiological disease activity and EDSS score at BL) were explored within groups by logistic regression analysis.

This study was approved by the local ethics board (number 2016.554) and complied with the declaration of Helsinki. All patients gave written informed consent.

## Results

**Figure 2.**
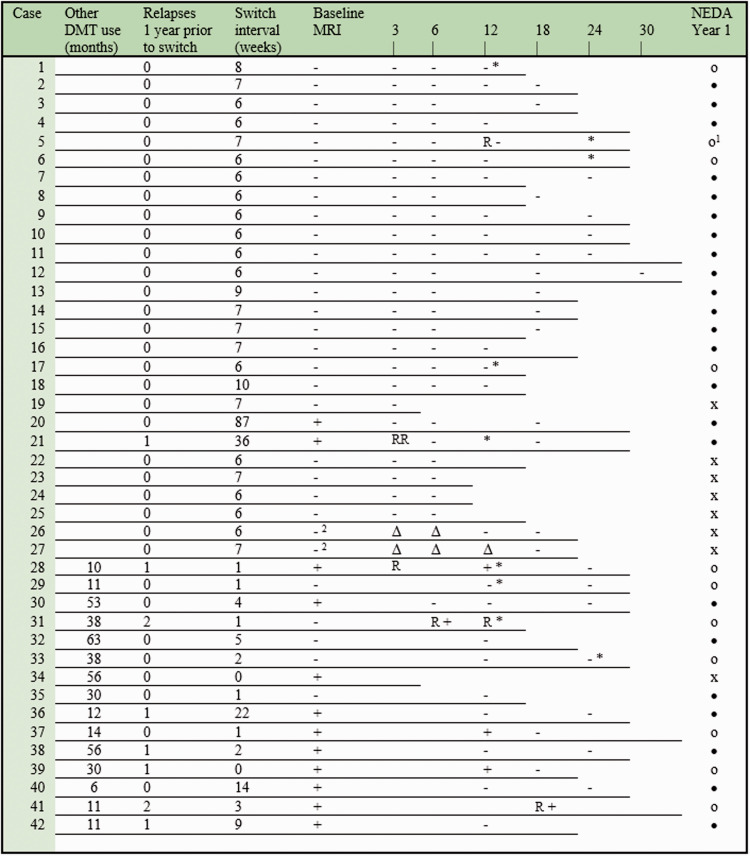
Clinical and radiological disease activity in relation to the last natalizumab dose and the first ocrelizumab dose (direct switchers, case 1 to 27) and other DMT use (indirect switchers). Case 28 to 38 used fingolimod, case 39 to 41 used daclizumab and case 42 used dimethyl fumaric acid. Each individual line represents the follow-up duration during ocrelizumab treatment. The result of each brain MRI scan is indicated with (+) in case of evidence of radiological activity and (−) in case of stability. Baseline MRI was performed at the last natalizumab dose (direct switchers) or within 3 months of ocrelizumab initiation (indirect switchers) as presented in Figure 1(a) and (b). Relapses are indicated with (R) and a ≥1 point increase in expanded disability status scale (EDSS) with (*). The last column illustrates no evidence of disease activity (NEDA) status for each case: (•) if it was maintained or established and (o) if it was lost. NEDA was assessed after 1 year of ocrelizumab treatment (therefore patients with shorter follow-up are indicated with (x)) and defined as no relapses, no evidence of radiological disease activity (excluding carry-over PML indicated by (Δ) and events that occurred during or within 3 months after long switch intervals between DMTs) and no EDSS progression (increase of 1.6, 1 or 0.5 point in case of baseline EDSS of 0, 1–5 or ≥5.5, respectively). ^1^Case 5 presented with new neurological symptoms (clinical localization in the thoracic spine) lasting for more than 24 hours confirmed by the treating neurologist, which complies to our study’s definition of an MS relapse. Due to the severity of the symptoms, the patient was treated with intravenous methylprednisolone (IVMP). Follow-up MRI of the brain and spine was performed 3 months later, which showed no MS activity compared to a brain MRI performed 6 months earlier and a spinal MRI performed 4 years earlier, which already showed a high lesion load in the suspected area. Given the treatment with IVMP, the timing and sensitivity of the spinal MRI, the clinical diagnosis was not rejected. ^2^Both PML cases showed one fast growing lesion on the brain MRI’s performed 5 weeks after the first ocrelizumab dose. In retrospect, these lesions were already present on the brain MRI’s performed during the switch interval, but not suspected of a PML lesion at that point.^5^

Forty-two JCV positive patients were included: 27 direct switchers and 15 indirect switchers (Table 1). Follow-up duration from last natalizumab dose or last other DMT dose until last follow-up during ocrelizumab treatment was 19.4 months [15–25] for direct switchers and 29 months [22–32] for indirect switchers. Clinical and radiological events of disease activity, NEDA status and adverse events are illustrated in Figure 2. During follow-up, 83% of direct switchers complied with NEDA criteria. Excluding patients with >12 weeks wash-out of natalizumab (case 20 and 21), 78% of direct switchers complied with NEDA criteria. Two male patients were diagnosed with carry-over PML 5 weeks (case 26) and 6 weeks (case 27) after the first ocrelizumab dose, with good recovery as described elsewhere. In retrospect, subtle signs of PML were present on the MRI scan performed prior to the switch in both patients.^5^ Respective natalizumab treatment durations were 6 and 10 years with JCV indices of 2.76 and 0.38 prior to switch. NEDA was found in 50% of indirect switchers after 1 year of ocrelizumab treatment.

**Table 1.** Clinical characteristics of direct (left column) and indirect (right column) switchers from natalizumab to ocrelizumab. Baseline characteristics are presented atthe last natalizumab dose. Numbers are indicated with (percentage), mean values with ± SD and median values with [interquartile range]. OCR FU1 indicates the second ocrelizumab 300 mg dose, OCR FU2 the first 600 mg dose and OCR FU3 the second 600 mg dose.

Baseline characteristics	Directswitchers (n = 27)	Indirectswitchers (n = 15)
Female individuals	17 (63)	9 (56)
Caucasian	26 (96)	15 (100)
Age (years)	39 ± 9.9	40 ± 11.3
Disease duration (years)	13.8 [11–18]	11.1 [9–17]
Years of natalizumab use^a^	3.5 [1.7–7.1]	4.7 [3.7–5.5]
EDSS^b^	3.0 [2.5–4.5]	3.5 [2.5–3.5]
Follow-up		
Switch interval (weeks)	6.4 [6.0–7.0]	6.5 [4.0–19.6]
Other DMT use (months)		29.5 [11–53]
- Fingolimod (n = 11)		38 [12–56]
- Daclizumab (n = 3)		11 [6–11]
- Dimethyl fumaric acid (n = 1)		29.5
Follow-up from last dose of previous DMT (months)	19.4 [15–25]	29 [22–32]
Number of ocrelizumab doses	5 [3–5]	5 [4–6]
JCV index		
- Last natalizumab dose	2.27 [0.86–3.38]^c^	2.20 [1.48–3.09]
- OCR FU1	1.46 [0.71–3.29]	2.13 [0.6–2.94]
- OCR FU2	1.34 [0.65–3.08]	1.56 [0.38–3.04]
- OCR FU3	1.10 [0.60–2.73]	
IgG (g/L)		
- Ocrelizumab screening	9.80 [8.2–10.6]	9.1 [7.9–10.0]
- OCR FU1	9.45 [7.6–10.5]	8.7 [7.8–10.6]
- OCR FU2	9.15 [7.9–10.2]	9.6 [8.1–10.9]
- OCR FU3	9.6 [7.9–10.1]	9.7 [8.1–11.0]
- OCR FU4	9.3 [7.8–10.1]	9.2 [8.0–11.0]
- OCR FU5	9.40 [7.1–10.8]	9.6 [8.0–10.7]
sNfL (pg/mL)		
- Last natalizumab dose	6.8 [5.5–7.8]	7.8 [6.1–11.0]
- OCR FU1	8.6 [5.5–10.8]	16.2 [9.6–28.2]
- OCR FU2	8.6 [5.1–12.0]	10.7 [8.1–16.8]
- OCR FU3	7.6 [6.4–11.0]	7.8 [5.2–11.8]
- OCR FU4	7.4 [5.1–8.8]	11.7 [7.5–14.5]

^a^In case of multiple NTZ treatment periods, only the duration of the last consecutive period is indicated.

^b^In patients who used other DMT in the period between natalizumab and ocrelizumab, median EDSS is calculated from visits closest to last administration of other DMT.

^c^JCV indices in direct switchers decreased significantly from last natalizumab to OCR FU3 (p = 0.000).

JCV indices in the total cohort decreased significantly during ocrelizumab treatment, while no significant changes occurred in IgG levels (Table 1). Indirect switchers had significantly higher median sNfL levels at OCR FU1 compared to direct switchers (p = 0.028). Median sNfL levels remained stable within each group over time. Within individuals, transiently increased sNfL levels correlated with PML (case 26 and 27), MS disease activity (case 21) and traumatic brain injury (case 7), and decreased over the course of ocrelizumab treatment. None of the covariates showed a significant odds ratio for not achieving NEDA status at year 1.

## Discussion

In this study, ocrelizumab treatment showed to be an effective alternative therapy for patients at risk of natalizumab associated PML. The rate of patients who established or maintained NEDA in our cohort is relatively lower in indirect switchers compared to direct switchers. As both groups were similar with regard to other risk factors for not achieving NEDA, this could mainly be due to the less effective treatment with other DMTs prior to ocrelizumab initiation. Despite our stringent surveillance protocol, 2 cases of carry-over PML occurred in the direct switch group. Switching to ocrelizumab did not cause PML in these cases, but we are unsure of the effect of B-cell depletion on the course of PML, which was mild in both patients.^5^ Although carry-over PML is rare, switching to DMT with long-lasting immunosuppressive effects poses a possible risk for JC-virus positive natalizumab-treated patients. The sNfL levels at ocrelizumab FU1 complied with the higher prevalence of disease activity on baseline MRI in indirect switchers, whereas the stable levels over time in both groups indicates effective disease suppression over the course of ocrelizumab treatment in accordance to previous studies.^6^

So far, other clinical studies on the efficacy and safety of switching patients from natalizumab to ocrelizumab are scarce, apart from one ongoing clinical trial with expected completion in June 2022 (Clinicaltrial.gov Identifier: NCT03157830). Three previous studies have comparable cohort sizes with a shorter follow-up and do not describe indirect switchers or biomarkers.^7–9^

Interestingly, JCV indices decreased during ocrelizumab treatment in this study. Since JCV carrier status is unlikely affected by switching to ocrelizumab, the decrease could reflect depletion of antibody producing cells induced by anti-CD20 therapy, although total IgG levels remained stable. A similar finding was previously reported in rituximab and fingolimod treatment,^10^ yet others reported no significant changes during ocrelizumab treatment.^11^ While awaiting future data, a low JCV index in MS patients treated with ocrelizumab should not necessarily be interpreted as JCV sero-negativity or a low PML risk category.

Limitations of our study include the small and differing sample sizes of the two groups and the fact that treatment decisions were based on individual preferences, although these reflect real-life practice.

In conclusion, our cohort supports the use of ocrelizumab after natalizumab to maintain suppression of disease activity. However, carry-over PML can still occur. This risk needs to be weighed against the risk of recurrence of disease activity when considering switching or bridging with other less effective DMTs.
